# Exploring the influence of takotsubo syndrome on oncologic patients’ mortality

**DOI:** 10.3389/fcvm.2022.1020078

**Published:** 2022-11-02

**Authors:** Giacomo Tini, Luca Arcari, Matteo Sclafani, Paolo Spallarossa, Giovanni Camastra, Allegra Battistoni, Camillo Autore, Massimo Volpe, Beatrice Musumeci, Pietro Ameri, Luca Cacciotti

**Affiliations:** ^1^Division of Cardiology, Department of Clinical and Molecular Medicine, Azienda Ospedaliera Universitaria Sant’Andrea, Sapienza University of Rome, Rome, Italy; ^2^Cardiology Unit, Madre Giuseppina Vannini Hospital, Rome, Italy; ^3^Cardiovascular Disease Unit, Istituto di Ricovero e Cura a Carattere Scientifico (IRCCS) Ospedale Policlinico San Martino, Genoa, Italy; ^4^Department of Internal Medicine, University of Genoa, Genoa, Italy

**Keywords:** takotsubo (stress) cardiomyopathy, cardio-oncology, cancer, CV health, heart failure

## Abstract

It has been reported that patients affected by takotsubo syndrome (TTS) with a concurrent diagnosis of cancer suffer from greater mortality as compared to their non-cancer counterpart. It remains unclear whether TTS worsens the prognosis of cancer patients as well. Aim of this study was to compare outcomes of cancer patients with and without TTS. We combined data from two independent cohorts: one consisted of a prospective multicentre TTS registry; the second cohort consisted of all oncologic patients from two Cardio-Oncology Outpatient Clinics, who did not have cardiovascular conditions at the time of the cardio-oncologic visit. From the TTS registry, we selected patients with cancer (cancer-TTS patients). Next, we matched these patients with those from the cardio-oncologic cohort (cancer non-TTS patients) in a 1:2 fashion by age, sex, and type and cancer staging. Study endpoint was all-cause mortality. Among 318 TTS patients, 42 (13%) had a concurrent diagnosis of cancer. Characteristics of cancer-TTS patients and of the 84 matched cancer non-TTS subjects were comparable with the exception of diabetes mellitus, which was more common in cancer non-TTS patients. All-cause mortality was similar between cancer-TTS and cancer non-TTS patients. At Cox regression analysis TTS was not associated with mortality (OR 1.4, 95% CI 0.6–3.3, *p* = 0.43). Our findings show that even in the presence of acute heart failure due to TTS, the prognosis of oncologic patients is driven by the malignancy itself. Our results may prove useful for integrated management of cardio-oncologic patients.

## Introduction

The profile of patients affected by takotsubo syndrome (TTS) is characterized by the presence of several cardiovascular and non-cardiovascular comorbidities, among which cancer is one of the most frequent (1, 2). It has been largely acknowledged that these comorbidities are the main drivers of prognosis in TTS, especially in the long-term (3). In particular, patients affected by TTS with a concurrent diagnosis of cancer have been reported to suffer from greater mortality, both in the short and long-term, as compared to their non-cancer counterpart (2, 4, 5). On the other hand, it remains unclear whether TTS worsens the prognosis of cancer patients.

## Methods

To assess the impact of TTS on cancer mortality, we combined the data from two independent cohorts. One consisted of 318 TTS patients enrolled in a prospective multicentre registry at S. Andrea Hospital, Sapienza University and Madre Giuseppina Vannini Hospital, both in Rome, Italy, since September 1, 2006 (6). Data were extracted from the registry on December 1, 2021. TTS was defined according to the European Society of Cardiology Heart Failure Association criteria (7). The second cohort was extrapolated from an all-comer population evaluated at two Cardio-Oncology Outpatient Clinics (IRCCS Ospedale Policlinico San Martino, Genova, Italy and S. Andrea Hospital, Sapienza University, Rome, Italy) since January 1, 2015. This latter cohort consisted of 1,024 oncologic patients referred for a baseline Cardio-Oncologic evaluation, who did not have cardiovascular conditions at the time of the cardio-oncologic visit.

From the TTS registry, we selected patients with either active cancer or cancer diagnosed within 5 years before the TTS event, hereafter defined as *cancer-TTS patients*. Next, we matched these patients with those from the cardio-oncologic cohort (*cancer non-TTS patients*) in a 1:2 fashion by age, sex and type and cancer staging (i.e., metastatic vs. non-metastatic).

Statistical analysis was performed with SPSS version 25. Continuous variables were compared by Student’s *t*-test; categorical variables by chi-squared test. The influence of TTS on all-cause mortality in cancer patients was assessed by means of Cox regression analysis and the Kaplan Meier method. Follow-up time was calculated from TTS or cancer diagnosis up to last evaluation or death, whichever came first. Secondarily, we also studied the relationship between cancer and all-cause mortality in the TTS cohort. For this analysis, follow-up began at the time of TTS diagnosis. A two-sided *p*-value < 0.05 was considered statistically significant. The study was conducted in compliance with the Declaration of Helsinki. As an observational retrospective study, it did not need full Ethics Committee approval; an informed consent for the use of medical data for research purposes was obtained from all participants.

## Results

Among 318 TTS patients (285 [90%] females, aged 72 ± 11 years, mean left ventricular ejection fraction: 40 ± 9%), 42 (13%) had a concurrent diagnosis of cancer. Types of cancer are reported in Table 1.

**TABLE 1:** Cancer details.

Type of cancer	*n*, %
Breast	12 (29%)
Colon	7 (17%)
Lung	5 (12%)
Gastric	4 (11%)
Adrenal gland	2 (5%)
Thyroid	1 (2%)
Ovarian	1 (2%)
Bladder	1 (2%)
Kidney	1 (2%)
Sarcoma	1 (2%)
Melanoma	1 (2%)
Hematologic*	6 (14%)

*Includes: chronic myeloid leukemia (2), multiple myeloma (1), Walderstrom macroglobulinemia (1), non-Hodgkin lymphoma (1), acute myeloid leukemia (1).

### Influence of takotsubo syndrome on oncologic patients’ mortality

**FIGURE 1:**
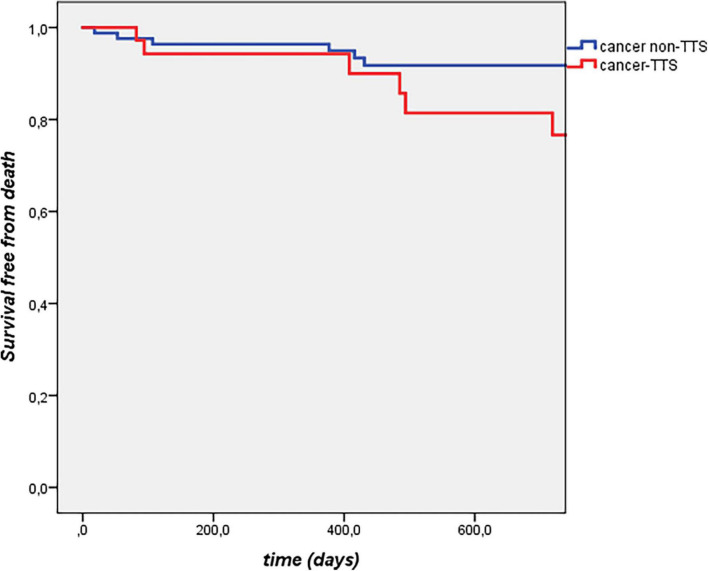
Kaplan Meier analysis for all-cause mortality.

Features of cancer-TTS patients and of the 84 matched cancer non-TTS subjects are reported in Table 2. Arterial hypertension, dyslipidaemia, and smoking were similarly distributed between the two groups, whilst diabetes mellitus was more common in cancer non-TTS patients. Length of follow-up was comparable in the two groups (median: 1.2 [0.2–3.3] vs. 1.9 [1.0–3.3] years, *p* = 0.08). All-cause mortality was similar between cancer-TTS and cancer non-TTS patients, with a 1-year incidence of 4.8 vs. 3.5%, respectively. At Cox regression analysis, TTS was not associated with mortality (OR 1.4, 95% CI 0.6–3.3, *p* = 0.43; *p* log = 0.43, Figure 1).

**TABLE 2:** Clinical features of cancer-TTS patients and matched cancer non-TTS ones.

	Cancer-TTS *n* = 42 (%)	Cancer non-TTS *n* = 84 (%)	*P*-value
Age, *mean* ± *SD*	72 ± 10	72 ± 10	-
Male sex	10 (24)	20 (24)	-
Hypertension	32 (76)	47 (57)	0.06
Dyslipidaemia	16 (38)	46 (55)	0.09
Diabetes mellitus	4 (10)	36 (43)	**<0.001**
Smoking	4 (10)	11 (13)	0.77

*P*-values < 0.05 are reported as bold.

### Influence of cancer on takotsubo syndrome patients’ mortality

There were no significant differences between TTS patients with and without cancer in terms of age, sex, cardiovascular risk factors, type of trigger, left ventricular ejection fraction at presentation and presence of atrial fibrillation (*p* > 0.05 for all, *data not shown*). During a median follow-up of 2.7 [0.5–6.5] years, 42 patients died, 10 (23.8%) with and 32 (11.6%) without cancer (*p* = 0.03). A diagnosis of cancer was associated with all-cause mortality at Cox regression analysis (OR 4.6, 95% CI 2.2–9.7, *p* < 0.0001), even after adjustment for age and sex (OR 3.1, 95% CI 1.3–6.9, *p* = 0.01).

## Discussion

Takotsubo syndrome does not influence the long-term prognosis of cancer patients. Our findings show that cancer-TTS and cancer non-TTS patients have a similar incidence of mortality, suggesting that even in the presence of an acute cardiovascular event (acute heart failure due to TTS), the prognosis of oncologic patients with active cancer is not substantially modified and that it is the malignancy that drives the outcome (8). In addition, our data confirm that TTS patients with cancer have a greater mortality as compared to TTS patients without, as already shown in previous studies (2, 4, 5). Long-term mortality in TTS is known to be burdened by non-cardiovascular comorbidities and, hence, non-cardiovascular deaths (2, 3, 9).

In real-world practice cardiovascular comorbidities or acute events are frequently perceived as debilitating when caring for a cancer patient (10). This may impact management and treatments (which may be halted, influencing the oncologic prognosis), and is particularly true in the case of heart failure (11), but has been postulated also in TTS (4, 12). Nevertheless, since the prevalence of malignancy in heart failure and in TTS is not low, as seen also in our cohort (11, 13, 14), and similarly is the prevalence of cardiovascular conditions and risk factors in oncologic patients (10), an integrated approach caring for bidirectional cardiovascular and oncologic needs appears fundamental, though frequently challenging to be pursued (15). In fact, dedicated guidelines for management of cancer patients in cardiovascular settings are lacking, and oncologic patients are often under-represented or not adequately phenotyped in cardiovascular clinical trials, and viceversa (11, 13, 16).

In this perspective, the fact that real-world cancer-TTS patients do not have a worse prognosis as compared to cancer non-TTS ones, might reassure oncologists who in their practice encounter patients experiencing, or who had experienced, an acute cardiovascular event as TTS. Moreover, given the clinical and epidemiological scenario depicted above, our findings further highlight the importance of collaboration between specialists, with the aim of not to undertreat cancer patients with cardiovascular comorbidities.

We acknowledge some shortcomings of our analysis. The registries from which the two matched groups have been extracted partly span different years. Moreover, the TTS one is a prospective registry, while cancer non-TTS patients were extracted from a retrospective cohort. Information about therapies and frailty or performance status in cancer patients were not available for all patients and thus were not included in the analysis. Finally, the two matched groups have a small size, possibly influencing statistical calculations. Our results should thus be considered hypothesis-generating, and we warrant replication in other cohorts. However, to our knowledge this is the first registry study comparing long-term outcome of TTS patients with cancer, with an oncologic counterpart, rather than with patients without cancer. Our considerations are not necessarily generalizable to any cardiovascular event outside the TTS context, and each one (i.e., acute myocardial infarction) likely deserves dedicated analyses. Nevertheless, it has frequently been reported that cardiovascular conditions in cancer patients are overlooked or undertreated (10). Given the significant improvements in cardiovascular and oncologic treatments, such a simplistic management is not anymore justified in clinical practice and needs to be addressed by the scientific community.

## Data availability statement

The original contributions presented in this study are included in the article/supplementary material, further inquiries can be directed to GT, giacomo.tinimelato@uniroma1.it; LA, luca.arcari88@gmail.com.

## Author contributions

GT and LA contributed to conception of the study. GT, LA, PS, BM, PA, and LC contributed to the design of the study. GT, LA, and MS organized the database. GT performed the statistical analysis and wrote the first draft of the manuscript. All authors contributed to manuscript revision and read and approved the submitted version.
